# Mandibular third molar protraction using a segmented arch and mini-implant anchorage: A case report

**DOI:** 10.1097/MD.0000000000046874

**Published:** 2025-12-26

**Authors:** Zhiqing Liu, Min Hu, Huichuan Qi, Jiyu Song, Jingzheng Yi, Mingjia Jiang, Yi Zhang

**Affiliations:** a Department of Orthodontics, Hospital of Stomatology, Jilin University, Changchun, Jilin, China; b Jilin Provincial Clinical Medicine Research Center of Orthodontics, Changchun, Jilin, China; c Western Dental.

**Keywords:** dental appliances, long-term stability, mandibular molar protraction, temporary anchorage device, tooth movement techniques

## Abstract

**Rationale:**

The absence of the mandibular second molar (M2) significantly compromises oral function and quality of life. Conventional prosthetic rehabilitation or autotransplantation presents notable limitations. Under the premise of efficiency and precision, orthodontic mesialization of the third molar (M3) using a localized appliance offers a promising, noninvasive alternative for tooth replacement.

**Patient concerns:**

A 20-year-old male patient with severe caries in the right mandibular M2 was referred by the Department of Endodontics and admitted to the Orthodontics Department at the Hospital of Stomatology, Jilin University, Changchun, Jilin, China, with the chief complaint of functional rehabilitation in the right mandibular region.

**Diagnoses:**

After conducting routine orthodontic examinations and analyses, the patient was diagnosed with skeletal Class III malocclusion and Angle Class I malocclusion. Additionally, the right mandibular M2 exhibited severe caries with pulp involvement and subgingival extension.

**Interventions:**

This case involved mesialization of the mandibular right M3 to substitute for the compromised M2, utilizing a segmental arch combined with a temporary anchorage device. Over 20 months, the M3 was mesialized by 11.7 mm, followed by 10 months of occlusal adjustment and 36 months of natural retention.

**Outcomes:**

The treatment successfully restored occlusal function on the right side. The M3 exhibited favorable root parallelism, preservation of the original occlusal plane, and maintenance of the facial profile.

**Lessons:**

The use of a segmental arch in combination with temporary anchorage devices enables effective mesialization of vertically positioned, two-rooted mandibular M3s. This approach offers a minimally invasive and low-risk alternative for the replacement of prematurely lost M2s in young adults.

## 1. Introduction

Tooth loss is often the ultimate consequence of progressive oral diseases, primarily caused by caries and periodontal disease.^[1,2]^ Additionally, trauma, developmental disorders, and eruption failure are significant etiological factors.^[3,4]^ Epidemiological studies indicate that the prevalence of untreated caries in permanent teeth peaks among individuals aged 20 to 24 years (36,319.99/10^5^).^[5]^ The mandibular second molar (M2), second only to the mandibular first molar in terms of caries susceptibility, is particularly vulnerable because of its complex anatomy and deep occlusal fissures and challenges in maintaining oral hygiene.^[6]^ Loss of the mandibular M2 not only disrupts the occlusal plane but also increases the functional load on the temporomandibular joint, thereby increasing the risk of temporomandibular disorders (TMDs). Prolonged dysfunction may further lead to a decline in oral health-related quality of life and psychosocial maladaptation.^[4,7,8]^ Consequently, restoring the mandibular M2 is crucial, especially in young adults. From a long-term prognostic and oral health perspective, conventional treatment options – such as dental implant restoration, fixed bridges, or removable prostheses – may not always be optimal.^[9,10]^ Although autotransplantation offers certain biological advantages, it is limited by donor tooth availability and procedural complexity.^[11,12]^ Orthodontic mesialization of the third molar (M3) to replace a missing M2 has emerged as a promising, noninvasive alternative. This approach restores function using the patient' s own dentition and avoids complications associated with prosthetic rehabilitation. Owing to its minimally invasive nature and preservation of natural tooth integrity, orthodontic mesialization of morphologically favorable mandibular M3s is increasingly favored by young adult patients.

However, conventional orthodontic approaches for M3 mesialization have inherent limitations. Prolonged treatment duration, difficulty in anchorage control, and unintended tooth movements – such as reciprocal distal drifting of adjacent teeth, unwanted tipping, or rotation – often compromise treatment outcomes. The advent of temporary anchorage devices (TADs) has revolutionized orthodontic biomechanics by providing absolute anchorage, thereby enhancing the predictability and efficiency of complex tooth movements.^[13,14]^ Nevertheless, extensive tooth movement over long distances requires a high level of three-dimensional control by the clinician. As a result, in current clinical practice, M3 mesialization is often performed in conjunction with comprehensive full-arch fixed appliance therapy.^[15]^ For patients with only M3 mesialization needs, the segmented arch can replace the full arch to improve the aesthetics and precision of treatment, reduce biological and economic costs for patients. However, the reduction in anchoring teeth will pose a greater challenge for clinicians.

This case demonstrates the complete mesialization of a mandibular M3 into the functional position of a previously extracted and nonrestorable M2 using TADs in conjunction with a segmental arch. A 36-month follow-up confirmed the long-term stability and functional success of this treatment approach.

## 2. Case presentation

### 2.1. Diagnosis and etiology

**Figure 1.**
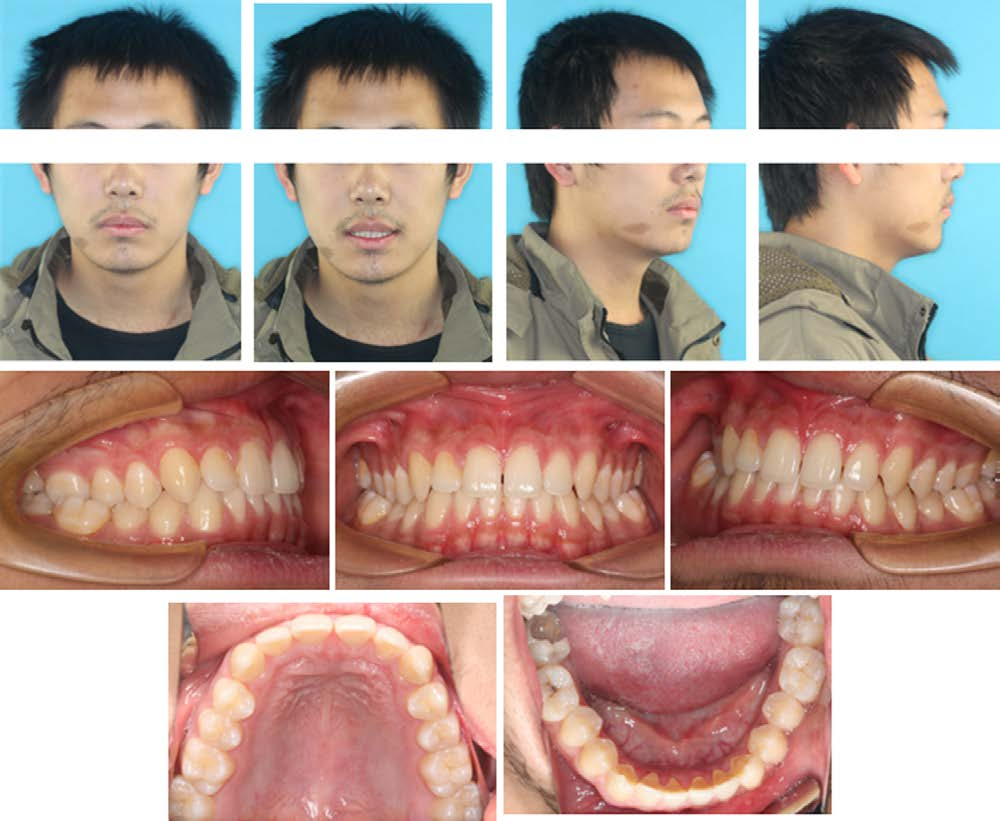
Pretreatment intraoral and extraoral photograghs.

**Figure 2.**
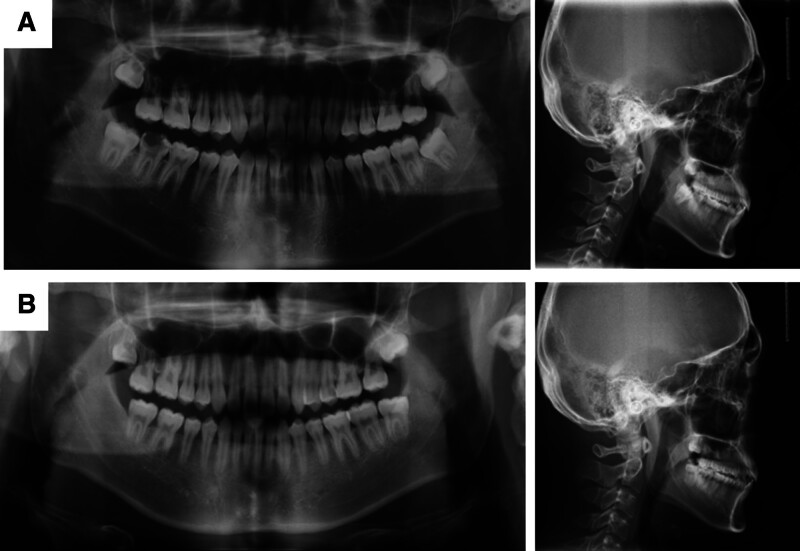
Pretreatment and posttreatment panoramic radiograph and lateral cephalogram.

A 20-year-old male presented to the Orthodontics Department at the Hospital of Stomatology, Jilin University, Changchun, Jilin, China, following a referral from Endodontics. His chief complaint involved functional rehabilitation of the mandibular right quadrant, specifically requesting mesialization of tooth #48 (FDI World Dental Federation, ISO-3950) to replace an unsalvageable tooth #47. Clinical and radiographic evaluation revealed that tooth #47 exhibited extensive caries involving the pulp, with subgingival destruction and nonrestorable crown morphology (Figs. 1 and 2A).

**Figure 3.**
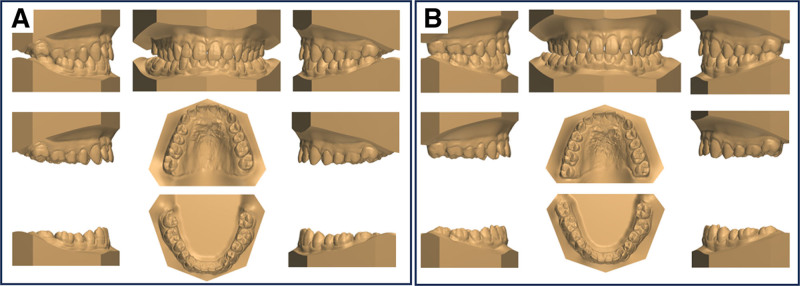
Pretreatment and posttreatment dental casts.

**Figure 4.**
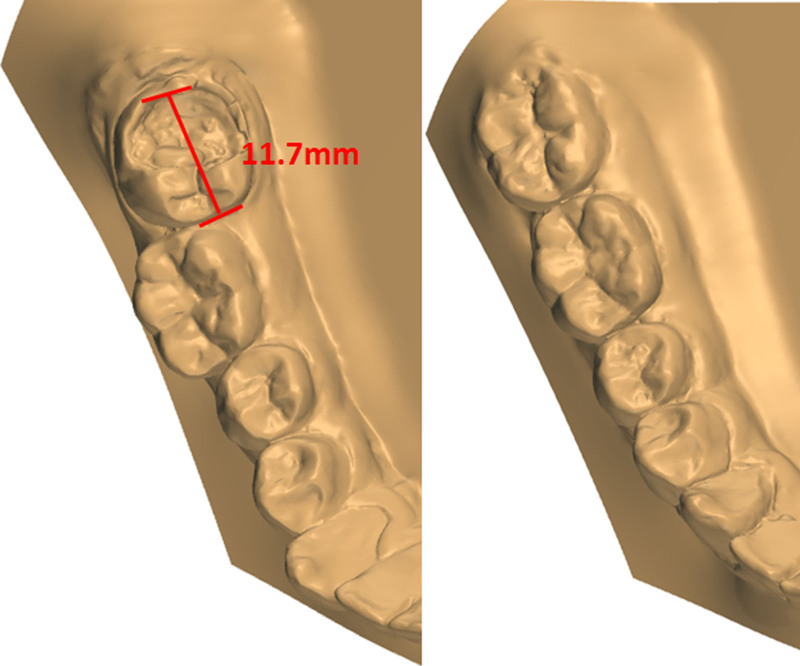
Close-up occlusal view of the right mandibular dental cast.

No facial asymmetry, optimal profile or proportionate chin morphology was evident in the frontal or lateral views (Fig. 1). Except for swallowing habits, no poor oral habits or symptoms of TMD were detected. On intraoral examination (Fig. 3), the patient showed Class I canine and molar relationships on both sides, but he had a bilateral posterior crossbite (BPXB) due to the noncorresponding dental arch width (Table 1). The overbite, overjet and midline were acceptable. Tooth #47 had extensive caries reaching the floor of the pulp chamber. The width of tooth #47 in the dental arch was 11.7 mm (Fig. 4). Periodontal examination revealed healthy conditions with no evidence of active pathology.

**Table 1:** Study cast analysis.

Anterior bolton analysis		Overall bolton analysis	
Upper 3–3 width	49.08	Upper 6–6 width	100.23
Lower 3–3 width	37.90	Lower 6–6 width	91.32
Ratio	0.772	Ratio	0.911

Arch-width analysis			
	Value	Difference	
Upper 3–3	36.14	8.98	
Lower 3–3	27.16		
Upper 4–4	39.62	5.27	
Lower 4–4	34.35		
Upper 6–6	51.45	−2.12	
Lower 6–6	53.57		

Radiological examinations revealed slight horizontal resorption of the alveolar bone, and tooth #47 revealed a shadow of caries without a shadow in the root canal. Tooth #48 had at least 2 roots (mesial root and distal root), both with fully formed apical foramina. And it had a pleasing shape without any significant tilting. No anomalies were detected in the condyle, maxillary sinuses, or jaws. The measurements of cephalometric and Tooth #48 are summarized in Table 2.

**Table 2:** Cephalometric measurements.

Measurements	Normal ± SD	Pretretment	Posttreatment
Skeletal			
SNA (°)	82 ± 3.5	77.1	76.3
SNB (°)	80.9 ± 3.4	78.6	77.7
ANB (°)	1.6 ± 1.5	−1.5	−1.5
FMA (MP-FH) (°)	22.9 ± 4.5	21.3	21.7
Occ plane to SN (°)	14.4 ± 2.5	21	20.2
Y-axis (SGn-SN) (°)	67 ± 5.5	70.8	70.9
Wits appraisal (mm)	-1 ± 1	-9.8	−8.4
Overbite depth indicator (ODI)	74.5 ± 5	62.8	63.5
Anteroposterior dysplasia (APDI)	81.4 ± 5	87.8	86.5
Combination factor (CF)	155.9 ± 2	154.6	154.2
Dental			
IMPA (L1-MP) (°)	95 ± 7	95.9	96.6
FMIA (L1-FH) (°)	65.7 ± 8.5	62.8	61.7
U1 - SN (°)	103.1 ± 5.5	98.2	99
L1 Protrusion (L1-APo) (mm)	2.7 ± 1.7	4.7	4.4
Tooth #48			
Angulation relative to occlusal plane (°)	–	81.9	85.9
Angulation relative to MP (°)	–	85.6	80.2

FMA = Frankfort mandibular plane angle; SD = Standard Deviation.

### 2.2. Treatment objectives

The primary objective of the treatment was to utilize the mesial movement of M3 or implant-supported prosthetics to restore the occlusal function of the edentulous area. The secondary objective was to correct BPXB and mild dental misalignment through full-arch orthodontic treatment and maxillary skeletal expansion devices, as well as to eliminate scattered spaces.

### 2.3. Treatment alternatives

The following treatment options considered are shown in Table 3. The patient selected the first option because of its reduced duration, accepting predetermined limitations including an unresolved crossbite and residual spacing. It was anticipated that within 2 to 3 years, a total of 11.7 mm mesial movement of tooth #48 and occlusal adjustment on the right side would be achieved. All potential risks, including root resorption, alveolar bone loss, and TAD failure, have been thoroughly explained to the patient. The patient fully understood the treatment plan and provided informed consent.

**Table 3:** Optional treatment plans.

Options	Orthodontics	Prosthodontic	Advantages	Disadvantages
**1**	a) Extract #47, expose #48; b) Segmented arch; c) Mini-implant to protract #48;	None	a) Low cost; b) Less surgical damage c) Preserve natural teeth;	Can' t correct transverse differentiation;
**2**	a) Extract #47, expose #48; b) Comprehensive orthodontic treatment; c) Potential MSE/MARPE to correct transverse differentiation; d) Protract #48 by mini-implant;	None	a) Preserve natural teeth; b) Correct transverse differentiation;	a) High cost; b) More surgical damage (MSE/MARPE);
**3**	None	a) Extract #47 & #48; b) #47 implants	a) Short treatment duration; b) Fewest follow-up visits	a) removing natural tooth; b) High cost c) High costs;

### 2.4. Treatment progress

**Figure 5.**
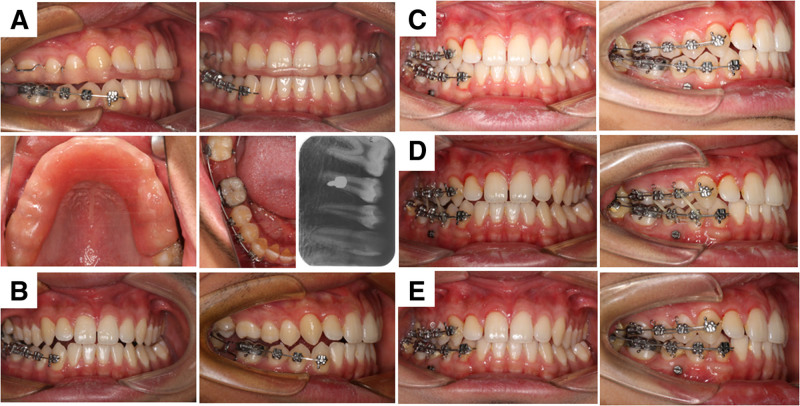
Orthodontic treatment process. (A) The bonding of brackets on the mandibular vestibular part, the placement of maxillary occlusal pads, and X-ray image of the implantation of mini-implants; (B) 4 mo later, 0.018 × 0.025-in ss with tip-back bends; (C) 20 mo later, #48 was continued to be pulled mesially, and bond brackets on the maxilla; (D) 25 mo later, the adjustment of the occlusion on the right side; (E) 30 mo later, on the day of appliance removal.

Upon finalization of the treatment plan, an orthodontic miniscrew (1.5 × 8 mm; Ningbo Cibei Medical Treatment Appliance Co., Ltd., Ningbo, China) was interdentally placed between tooth #44 and #45. Surgical extraction of tooth #47 and fenestration-assisted exposure of tooth #48 were performed. A segmented vestibular fixed appliance (Hangzhou West Lake Biomaterials Co., Ltd., Hangzhou, China) was bonded to teeth #43 to #45, complemented by molar tubes (Hangzhou West Lake Biomaterials Co., Ltd., Hangzhou, China) and lingual buttons on teeth #46 and #48. A 0.014-inch nickel–titanium archwire was used for initial alignment. A prefabricated maxillary occlusal splint was used to mitigate occlusal interference during mandibular molar mesialization (Fig. 5A).

Four months later, the mandibular archwire was upgraded to a 0.018 × 0.025-inch stainless steel segment. The previously placed miniscrew served as direct anchorage for controlled mesial traction of tooth #48. The mesial traction force was applied with elastic chain, with a force of approximately 200 grams between #48 and miniscrew. A tip-back bend, labial root torque, and supplemental lingual elastic traction were incorporated to counteract adverse crown displacement during translocation. Concurrently, figure-eight ligation was applied to teeth #44 to #46 to reinforce anchorage integrity (Fig. 5B). During this period, the patient returned for follow-up appointments approximately every 4 to 6 weeks for elastic chain replacement and adjustment.

Sixteen months later, the molar had achieved optimal positional alignment. However, tooth #17 presented occlusal interference, resulting in suboptimal right posterior functional relationships. Consequently, the right maxillary quadrant was integrated into the treatment protocol. Brackets were bonded to the maxillary teeth, and a 0.014-inch nickel–titanium archwire was added. A supplementary orthodontic miniscrew (1.5 × 8 mm; Ningbo Cibei Medical Treatment Appliance Co., Ltd., China) was placed interdentally between tooth #15 and #16 to enhance anchorage control (Fig. 5C).

Four months later, following progression to a 0.018 × 0.025-inch stainless steel archwire, a lingual crown torque was applied to tooth #17. Vertical elastics were engaged between teeth #14 to #15 and teeth #44 to #45 to address anterior open bite and occlusal plane discrepancies (Fig. 5D). Upon achieving stable occlusion and midline alignment, all appliances were debonded and replaced with a Begg retainer (Fig. 5E).

### 2.5. Treatment results

**Figure 6.**
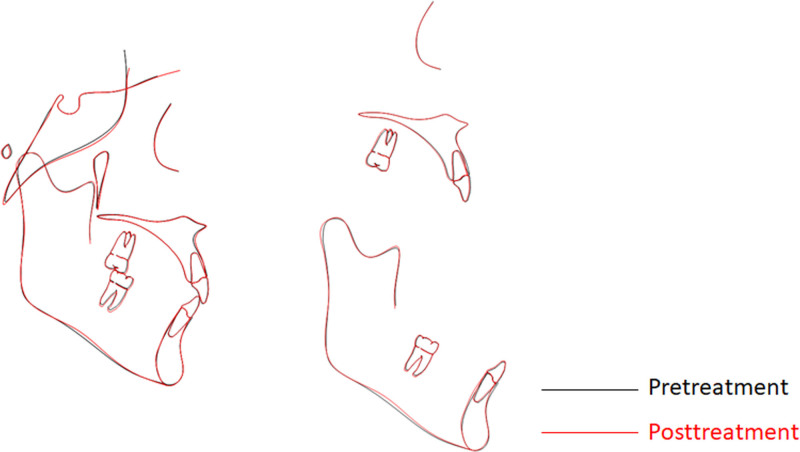
Cephalometric superimpositions between pretreatment (black) and posttreatment (red).

**Figure 7.**
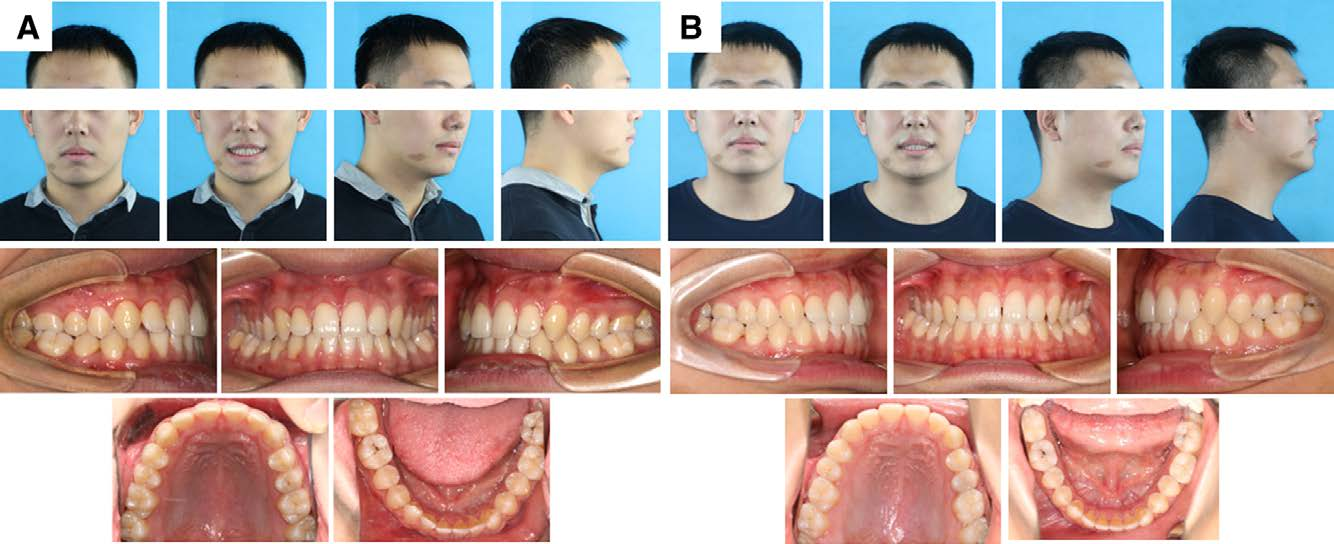
Intraoral and extraoral photographs at posttreatment (A) and 36-mo follow-up (B).

The total treatment duration for the patient was 30 months, comprising 2 phases: 20 months for controlled mesialization of tooth #48 and 10 months for occlusal refinement of the right maxillomandibular quadrant. The posttreatment photos and dental casts (Figs. 3 and 7A) revealed that the originally severely carious tooth #47 had been replaced. The right posterior alignment and occlusion were optimal. The pretreatment anterior spacing and molar crossbite persisted, which was consistent with the patient-accepted outcomes. The facial profile remained unchanged. Superimposition tracings (Fig. 6) and cephalometric analysis (Table 2) demonstrated no significant changes in the patient' s occlusal plane, dental axes, or occlusal relationships. Panoramic radiography confirmed adequate root parallelism and 11.7 mm of bodily mesial movement of tooth #48 (Fig. 2B), with negligible root resorption observed. At the 36-month follow-up, the position of tooth #48 and the occlusion remained stable (Fig. 7B). Throughout the active orthodontic treatment phase and subsequent retention phase with the Begg retainer, the patient exhibited excellent compliance. Moreover, he expressed a high level of satisfaction with the treatment outcomes, particularly regarding aesthetics and occlusal function.

## 3. Discussion

Endodontic and restorative treatments for terminal molars in the dental arch present significant clinical challenges. Although these interventions can be predictable, they are often associated with inherent biological and biomechanical complications, necessitating meticulous long-term maintenance protocols.^[6,16]^ Moreover, prosthetic approaches alone often fail to preserve the alveolar bone volume in edentulous regions over time. Orthodontic space closure via mesialization capitalizes on the natural mesial drift potential and periodontal ligament architecture of the M3, promoting physiological adaptation. Compared with prosthetic replacements, this approach may result in superior long-term periodontal health. Importantly, successful orthodontic M3 mesialization not only restores posterior occlusion but also provides positive stimulation to the alveolar bone in the molar region, thereby mitigating postextraction ridge resorption. However, extensive molar mesialization deviates from conventional principles of adult orthodontics, which generally favor minor tooth movements because of the reduced adaptability of mature periodontal tissues and alveolar bone. Molars, with their large roots and broad periodontal ligament areas, located at the distal end of the arch, require substantial force for movement and pose considerable control challenges. Therefore, precise treatment planning, advanced biomechanical techniques, and dependable anchorage strategies are essential for success.

The patient in this case presented with posterior crossbite attributed to inherent skeletal discrepancies between the maxilla and mandible (Table 1). Clinically, posterior crossbite may manifest unilaterally or bilaterally, with unilateral presentations being more common.^[17,18]^ Posterior crossbites can induce alterations in masticatory muscle function, increase the frequency of reverse chewing cycles, potentially increase the risk of developing TMD, reduce masticatory efficiency, and initiate a cascade of periodontal deterioration.^[19–21]^ However, it is important to note that electromyographic activity differences associated with posterior crossbite are not necessarily considered pathological, and the causal relationship between posterior crossbite and TMD remains controversial. Notably, compared with unilateral posterior crossbites, BPXB – when occlusal function is within acceptable parameters – may activate adaptive remodeling of the stomatognathic system via symmetrical stress distribution. This has been associated with improved temporomandibular joint stability and better periodontal outcomes than those observed with unilateral involvement.^[17,22]^ Therefore, for this BPXB patient without TMD or masticatory muscle dysfunction, adopting a localized orthodontic approach is reasonable. Additionally, the patient' s slight dental spacing, mainly located between the maxillary central incisors, neither impacts function nor aesthetics, while also facilitating oral hygiene maintenance. The minimally invasive conservative philosophy allowed us to preserve the patient' s original anterior crossbite and minor dental spacing.

Given the scientific feasibility of the proposed treatment plan and the patient' s lack of strong desire for comprehensive orthodontic therapy, the clinical focus was placed on the management of the severely carious molar through mesialization of tooth #48 as a replacement. The center of resistance for molars is typically located 1 to 2 mm apical to the root furcation, while the force vector provided by TADs needs to be loaded via brackets positioned on the buccal side of the crown. Due to the reason, the mesialization process often results in undesirable mesial tipping, lingual rotation, and slight lingual inclination of the molars. To minimize excessive stress on adjacent structures, a T-loop was employed to achieve stress interruption. The distal arm of the T-loop was bent with a tip-back and torque component to counteract the lingual inclination and mesial tipping generated during molar mesialization. Compared with conventional closing loops and Albert loop,^[15,23]^ the T-loop significantly increases the effective length of the archwire, allowing for more controlled and physiologic force levels.^[24]^ Moreover, to address the potential rotational instability associated with the flexibility of the T-loop and to compensate for diminished control over the distal segment of the archwire, lingual buttons were bonded to the lingual surfaces of tooth #46 and #48 to deliver an active counterrotational movement.

**Figure 8.**
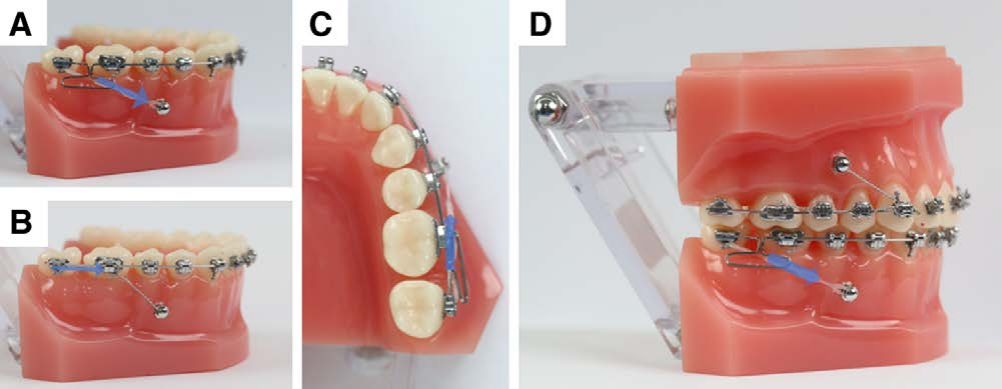
Schematic of anchorage modalities and loading mechanics for mandibular molar mesialization. (A) Direct anchorage; (B) indirect anchorage; (C) mini-implant loading pattern during molar protraction; (D) combined direct/indirect anchorage application in the case.

In recent years, the widespread use of TADs has provided reliable anchorage for molar mesialization. TAD-assisted molar movement strategies are broadly classified into direct and indirect anchorage approaches (Fig. 8A and B). Direct anchorage typically involves a TAD directly connected to the target tooth via elastomeric chains or NiTi coil springs to generate mesializing forces and is currently the more commonly adopted method.^[15,25,26]^ Owing to anatomical constraints, however, the force vector often forms an angle with the desired direction of molar movement and may impinge upon the soft tissue. To avoid such complications and ensure linear force transmission, TADs are usually placed between the mandibular premolars (Fig. 8C), where force can be delivered along a straight path with minimal lingual side effects on the anterior segment. Indirect anchorage, by contrast, refers to connecting the TAD to an anchorage tooth or directly to the archwire via a rigid structure, forming a stabilized unit.^[8,27]^ While this approach aligns the force vector more closely with the direction of molar movement, direct anchorage provides more efficient force transmission by eliminating intermediate structures, thus reducing unintended tooth movement and mechanical losses. These advantages that make it especially suitable for long-distance molar mesialization. In this case, we adopted a direct anchorage strategy by placing a TAD between the mandibular premolars to exert consistent mesializing force on tooth #48. For torque control of the maxillary molars, where available anchorage teeth are limited, we employed an indirect anchorage system by placing buccal TADs connected rigidly to the anchorage teeth to minimize unwanted movement of the adjacent dentition (Fig. 8D). Before initiating treatment, the patient was fully informed about the potential risk of TADs failure. In this case, neither of the 2 TADs loosened or dislodged during the entire course of treatment, largely due to a combination of favorable factors: the patient' s age, excellent oral hygiene, and the application of appropriate force levels during orthodontic treatment. A systematic review also noted that these factors are critical for ensuring the long-term stability of TADs.^[28]^

Recent clinical reports^[4,15,26]^ on TAD-assisted molar protraction indicate a molar movement rate of approximately 0.35 to 0.80 mm/month, with a total tooth displacement of 6 to 11 mm over treatment periods ranging from 12 to 30 months. In the present case, a molar movement rate of 0.69 mm/month was achieved, which falls at the higher end of the reported range. Moreover, the total mesial movement of 11.7 mm is clinically considered challenging, particularly as it exceeds the commonly cited 10 mm threshold in the literature.^[13,26]^ This favorable outcome is attributable to meticulous biomechanical planning and consistent force control throughout the treatment. Nevertheless, several key considerations arise with such extensive tooth movement. During treatment, it is crucial to closely monitor gingival status, tooth mobility, and patient feedback at each follow-up visit. Particular attention should be paid to alveolar bone remodeling and potential root resorption, both of which are major concerns for clinicians and patients following long-distance tooth movement.^[3,25]^ Lastly, the retention phase is paramount for maintaining stability. In this case, the patient' s excellent compliance with the Begg retainer over 36 months, in combination with a favorable cusp-fossa occlusion, contributed to the favorable long-term outcome observed.

In this case, to expedite the mesial movement of tooth #48, we extracted tooth #47 at the initial treatment phase. This approach fully leveraged the biological window presented by the extraction socket' s healing process, thereby creating favorable conditions for subsequent molar mesialization. Yuan^[29]^ further substantiated this strategy by demonstrating that planned extraction of an adjacent tooth, followed by orthodontic movement, can maximize preservation of the buccolingual alveolar bone and thus facilitate tooth passage through the extraction site, ultimately improving overall treatment efficiency. Although surgical adjuncts are widely regarded as the most effective means of accelerating tooth movement,^[15,30]^ the removal of tooth #47 at the start of therapy in the present case yielded a satisfactory tooth movement rate through natural healing while maintaining alveolar bone width. Considering the invasive nature of surgical interventions and their associated risks relative to any additional benefits for this particular patient, we elected not to employ auxiliary surgery.

## 4. Conclusion

The combination of a segmental arch with TADs enables efficient mesialization of upright, double-rooted mandibular M3s, providing a minimally invasive and low-complication alternative for young adults with early loss of the M2.

## Acknowledgments

“We are grateful to the patient for his cooperation during the treatment and the permission to use his clinical data.” We also appreciate all colleagues in the orthodontic department for their cooperation during the patient' s treatment process.

## Author contributions

**Formal analysis:** Mingjia Jiang, Yi Zhang.

**Funding acquisition:** Yi Zhang.

**Methodology:** Min Hu, Huichuan Qi.

**Project administration:** Yi Zhang.

**Resources:** Yi Zhang.

**Software:** Huichuan Qi.

**Supervision:** Jiyu Song.

**Validation:** Jiyu Song, Jingzheng Yi.

**Visualization:** Jingzheng Yi.

**Writing – original draft:** Zhiqing Liu.

**Writing – review & editing:** Zhiqing Liu.
